# MicroRNA-106a Provides Negative Feedback Regulation in Lipopolysaccharide-Induced Inflammation by targeting TLR4

**DOI:** 10.7150/ijbs.33432

**Published:** 2019-08-22

**Authors:** Jing Yang, Yu Chen, Kangfeng Jiang, Yaping Yang, Gan Zhao, Shuai Guo, Ganzhen Deng

**Affiliations:** Department of Clinical Veterinary Medicine, College of Veterinary Medicine, Huazhong Agricultural University, Wuhan 430070, People's Republic of China. College of Veterinary Medicine, Huazhong Agricultural University, Wuhan, 430070, People's Republic of China.

**Keywords:** ALI, miR-106a, NF-κB, TLR4

## Abstract

Acute lung injury (ALI) is a common clinical disease with high incidence and mortality rate, which is characterized by severe inflammatory response and tissues damage. MicroRNAs (miRNAs) have been regarded as novel regulators of inflammation, and play an important role in various inflammatory diseases. However, it remains unknown whether the regulatory mechanisms mediated by miR-106a is involved in LPS-induced ALI. In this study, we found that expression of miR-106a was significantly decreased in lung tissues of ALI mice and LPS-stimulated macrophages. We also revealed that over-expression of miR-106a significantly decreased the production of pro-inflammatory cytokines, including IL-1β, IL-6 and TNF-α, whereas this effect was reversed by the inhibition of miR-106a. Moreover, miR-106a inhibits NF-κB activation by targeting TLR4 expression. We further demonstrated that miR-106a inhibited TLR4 expression via binding directly to the 3'-UTR of TLR4. Taken together, the results of the present study illuminated that miR-106a is a negative feedback regulator in LPS-stimulated inflammation through TLR4/NF-κB signaling pathway.

## Introduction

Inflammation is a complex biological process with coordination of various organism functions to clean the pathogenic microorganism 1, 2. Acute lung injury (ALI) is a type of clinically uncontrollable inflammatory responses in lung tissues caused by various internal and external factors, such as major trauma, sepsis and pneumonia 3. ALI is characterized by the extensive neutrophils infiltration and over-expression of inflammatory mediators. Finally, these inflammatory mediators interact with each other in a complicated manner that results in diffuse pulmonary edema, organ injury and even respiratory distress 4, 5. Therefore, it is necessary to further clarify the mechanism of pulmonary inflammation development.

It is generally accepted that ALI has intrinsic correlation with Gram-negative infections in some cases 6. Lipopolysaccharide (LPS), one of the best researched immunostimulatory components of Gram-negative bacteria 7, is a potent activator of various mammalian cell types and plays a vital role in inflammatory responses 8. Previous studies have determined that LPS could induce ALI in mice by activating TLR4/NF-κB signaling pathway, which may be the key target to result in inflammatory response and organ injury 9. Correspondingly, the expression levels of multifarious mediators particularly pro-inflammatory cytokines, such as interleukin-1β (IL-1β), interleukin-6 (IL-6) and Tumor Necrosis Factor-α (TNF-α), is significantly increased 10. These cytokines are involved in the innate immune response and further cause severe damage to the lung tissues, which ultimately leads to ALI 11, 12. Therefore, the development of ALI induced by LPS can be inhibited by blocking TLR4-mediated NF-κB signaling pathway.

MicroRNAs (miRNAs), approximately 22 nucleotides, are a class of evolutionarily highly conserved, endogenous, small and non-coding RNAs, which function in mRNAs silencing and are mainly involved in the post-transcriptional gene regulation 13. In animals, miRNAs are processed from long primary transcripts (pri-miRNAs) through an approximately 70 base-pairs hairpin precursor step (pre-miRNAs) into the mature forms by continuous cutting with Drosha and Dicer enzymes 14, 15. Once in mature form, miRNAs direct the silencing complex (RISC) to degrade mRNA or hinder its translation by pairing with 3'-untranslated regions (3'-UTRs) of target mRNAs 16. Previous researchers have demonstrated that the significant effect of various miRNAs on the degree of inflammatory response is through targeting signal transduction proteins or directly targeting encoding pro-inflammatory cytokines' mRNAs 17. In addition, plenty of studies have identified that some miRNAs are likely involved in modulating signaling transduction during immune and inflammatory responses via Toll-like receptors (TLRs) pathway 18, 19. miR-106a is a member of miR-17 precursor family, which decreases expression by LPS stimulation 20. Recently, it has been reported that miR-106a is connected with tumor growth and immune response as well as suppressing TNF-α-induced transcription of pro-inflammatory genes 21-23. miR-106a is also down-regulated in T helper 17 cell differentiation 24. More importantly, miR-106a is also involved in an ozone-induced lung inflammatory feedback in acute mouse model 25. These findings prompted us to fully investigate the role of miR-106a in inflammatory responses to LPS both *in vivo* and *in vitro*.

Here, we found that the expression of miR-106a was prominently down-regulated in lung tissues of ALI mice and in macrophages, which both developed by administration of LPS. We also showed that transfection with miR-106a mimics resulted in a down-regulation in NF-κB activation and in pro-inflammatory cytokines including IL-1β, IL-6 and TNF-α, whereas miR-106a inhibitors transfection led to an up-regulation in pro-inflammatory cytokines. Further characterization of miR-106a demonstrated that miR-106a bound directly to the 3'-UTR of TLR4 to suppress TLR4 expression. Our findings revealed that miR-106a might be a crucial negative regulator of the immune response and provide new ideas for the therapeutic accesses to some inflammatory diseases including ALI.

## Methods and materials

### Animals and induction of ALI

Male BALB/c mice (8 weeks of age, 30-35g weight) purchased from Experimental Animal Center of Huazhong Agricultural University (Wuhan, China) were used. All animals were retained under standard conditions of 22℃ ± 1℃ and a 12-h light/12-h dark cycle, and were supplied food and water ad libitum. The Ethical Committee on Animal Research of Huazhong Agricultural University approved all animal experiments, which were in accordance with guidelines provided by the National Institutes of Health Guide for the Care and Use of Laboratory Animals. The mice were randomly divided into two groups, Control group (n=12) and LPS group (n=12). The methods for developing the LPS-stimulated ALI model were described previously 26. In LPS group, mice were intratracheally administered with 1,000 µg/ml LPS (Escherichia coli 055:B5) at the dose of 15 mg/kg body mass, while mice in control group were treated with equal dose of PBS. After 24 h, we used the sodium pentobarbital to euthanize mice, and the lung tissues were obtained. Three mice in each group were selected to measure the wet and dry weight of the lungs, three other mice were used to evaluate histological changes, and the remaining six mice were used to perform molecular biological analyses.

### Histological analysis

The lung tissues were harvested and fixed in 4% paraformaldehyde for histological analysis. After 24 hours, the lung tissues were embedded in paraffin, cut into 4-µm thick sections, sliced, and then stained with hematoxylin and eosin (H&E). Subsequently, pathological changes of lung tissues were observed under a light microscope.

### Lung wet to dry weight (W/D) ratio

The W/D ratio of lung tissues was calculated to measure the severity of pulmonary edema. The lung tissues from each group were reaped, rinsed briefly by PBS, and then weighed to acquire the 'wet' weight. Subsequently, the lung tissues were incubated at 80℃ for 24 hours to obtain the 'dry' weight. The lung W/D ratio was calculated through dividing the wet weight by the dry weight.

### The myeloperoxidase (MPO) assay

MPO is a functional and activation marker of neutrophils and macrophages, the level of MPO activity was measured to predict the early inflammatory diseases 27. To analyze the MPO activity, lung tissues were collected and homogenized with reaction buffer (w/v, 1/19). The MPO activity was performed using an MPO determination kit (Jiangcheng company, Nanjing, China) with a spectrophotometry at 460 nm according to the manufacturer's protocols.

### Cell culture

RAW264.7 cells (the murine macrophage cell line) and HEK293T cells(the human embryonic kidney cell line) were purchased from American Type Culture Collection. RAW264.7 cells were grown in DMEM (Invitrogen, Carlsbad, CA, United States) with 10% FBS (Invitrogen, Carlsbad, CA, United States), streptomycin /penicillin at 37 ℃ with 5% CO_2_ incubator. HEK293T cells were cultured in DMEM supplemented with 10% FBS at 37 ℃ in 5% CO_2_ incubator.

### Cell viability assay

Cell viability was determined using a Cell Counting Kit-8 (CCK-8) assay kit purchased from Beyotime (Shanghai, China) to detect the effect of LPS treatment. RAW264.7 cells were plated at a density of 4×10^4^ cells/well in 96 well plates and cultured approximately 1 h. After the cell adherence, the cells were treated with or without LPS for 0, 6, 12 or 24 h. Then, 10 μl of CCK-8 solution was added into each well for another 3 h at 37℃ in 5% CO_2_ incubator. Finally, the optical density (OD) of the macrophages in each well was measured at 450 nm using a microplate reader (Bio-Rad Instruments, Hercules, CA, United States).

### Computational predication of the miRNA targets

In order to further analyze the effects of miRNA-106a, two computational approaches, Targetscan (www.targetscan.org) and miRanda (www.microrna.org) were used to predict the targets of miRNA-106a in the Toll-like receptors (TLRs) signaling pathways. mirSVR, an algorithm, can be used to score and rank the efficiency of miRanda-predicted micro RNA target sites. The scores can be regarded as empirical probability of a single gene down-regulation by a specific micro RNA 28. The targets predicted by both modules and included receivable mirSVR downregulation scores were selected for additional studies.

### Enzyme‑linked immunosorbent assay (ELISA)

RAW264.7 cells were transfected with miRNA-106a mimics or inhibitors or the respective controls for 24 h, and then stimulated with 2 μg/mL LPS for 24 h. To determine the release of pro-inflammatory cytokines, the levels of IL-1β, IL-6 and TNF-α in the lung tissues and cell supernatants were measured with ELISA kits (R&D Systems, Inc.,Minneapolis, MN, USA), according to the manufacturer's protocols.

### RNA extraction and Quantitative real-time PCR

In accordance with the manufacturer's recommendation, total RNAs from lung tissues and cells were extracted using the TRIzol reagent (Invitrogen, United States). OD values were used to assess the concentration and purity of extracted RNA at 260 and 280 nm using Q5000 (Quawell Technology, United States). The samples' ratios of OD260 to OD28 were between 1.9 and 2.0. For miRNA analysis, M-MLV reverse transcriptase with a special reverse transcription primer for miRNAs was performed to synthesize the cDNAs. PCR was analyzed using a miRNAs real-time PCR kit (GenePharma, Shanghai, China) according to manufacturer's instructions. The primers for miR-106a and U6 snRNA were purchased from GenePharma (**Table 1**). For mRNA analysis, a PrimeScript RT reagent kit (Takara, Dalian, China) was used to synthesize cDNA in accordance with the manufacturer's protocols. PCR was executed using SYBR Green plus reagent kit (Roche, Basel, Switzerland) following the manufacturer's instructions. The relative expression levels of miR-106a and each mRNA were normalized to U6 snRNA and GAPDH with 2^-△△Ct^ method as described previously 29. The primers for qPCR are listed in **Table 2**.

### Western blot analysis

To prepare total protein, post-transfection RAW264.7 cells were collected and lysed with a lysate containing phosphatase inhibitor, then centrifuged at 4℃ and 12,000 rpm for 15 min. The obtained protein concentrations were determined using the Pierce BCA Protein Assay Kit. Subsequently, protein samples (40μg per well) were separated by 10% SDS-PAGE gel, transferred to polyvinylidene difluoride (PVDF) membranes, and blocked in skimmed milk for 2 h, then probed overnight with primary antibodies (1:1000) at 4℃. Membranes were washed and incubated with secondary antibodies (1:4000) for 1 h at 25℃, and visualized using enhanced chemiluminescence.

### Luciferase Reporter Assay

In order to explore the function of miR-106a in the LPS-stimulated NF-κB activation, a NF-κB luciferase reporter assay was performed, as described previously 30. In brief, macrophages were co-transfected with NF-κB luciferase plasmid and miR‑106a mimics along with indicated controls for 24 h. Then, the RAW264.7 cells were induced with 2 μg/mL LPS for another 24 h. The macrophages were lysed, and the luciferase activity was measured by the Dual-Luciferase Reporter Assay System (Promega) in accordance with the manufacturer's protocols.

To determine whether miR-106a targets TLR4 directly, the predicted and mutated sequences of 3'-UTR of TLR4 were amplified and cloned into a psiCHECK^TM^-2 vector (Promega, Madison, WI, United States) to generate a Luc-WT-3'-UTR vector or Luc-MUT-3'-UTR vector. For the luciferase assay, HEK293T cells were seeded into12‑well plates at a density of 2×10^5^ cells per well, and co-transfected with the Luc-WT-3'-UTR vector or Luc-MUT-3'-UTR vector and miR-106a mimics or controls using Lipofectamine 2000 (Invitrogen; Thermo Fisher Scientific, Inc.). After 24 h of transfection, the luciferase activities were measured by the Dual-Luciferase Reporter Assay System according to the instructions of manufacturer.

### Immunofluorescence Staining

RAW264.7 cells (2 × 10^5^ cells/mL) were seeded onto a 6-well plates. Immunofluorescence staining was performed, after cells were washed with PBS, fixed with 4% paraformaldehyde for 20 min, and blocked with PBS containing 0.3% Triton X‑100 and 10% BSA at room temperature for 1 h. Subsequently, cells were incubated with rabbit anti-p-p65 antibody and anti-TLR4 antibody overnight at 4℃, then cells were washed and followed by incubation with FITC-labeled secondary antibodies in the dark for 1 hat room temperature. Nuclei were stained using DAPI for 15 min in the dark, and fluorescent images were observed using an inverted fluorescence microscope.

### Statistical analysis

Statistical analysis was performed using The SPSS software 16.0 (SPSS Inc.). Quantitative datas are expressed as the mean ± SEM. Statistical analysis was performed using independent-samples t-tests between two groups. The values of p<0.05 were considered to indicate a statistically significant difference. All experiments were performed in triplicate and repeated three times.

## Results

### The expression of miR-106a is suppressed in the lung tissues of LPS-induced ALI mice

**Figure 1:**
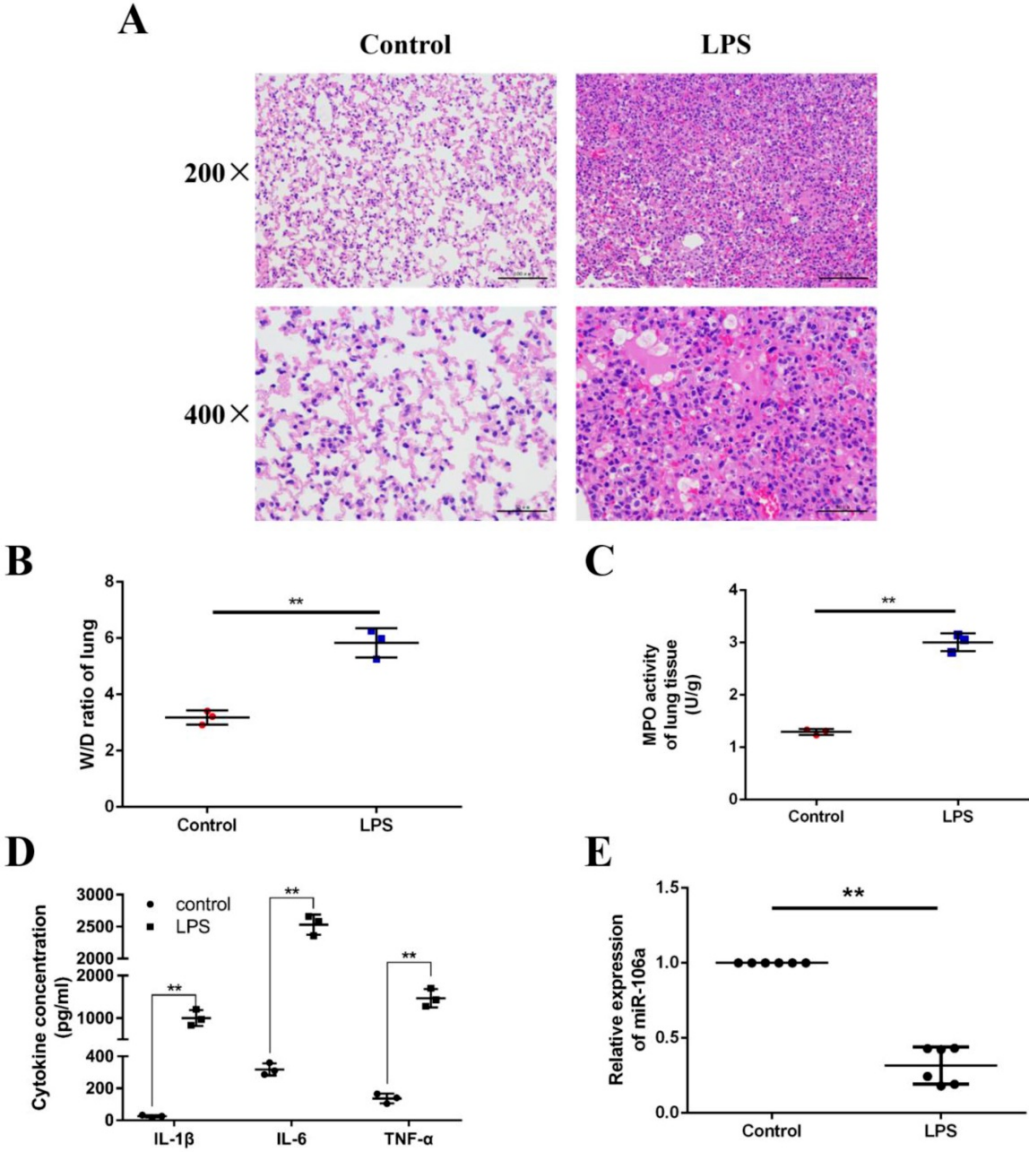
** miR-106a is down-regulated in the lung tissues of LPS-induced ALI mice. (A)** Histopathological analysis of lung tissues. Mice were intratracheally administered with LPS for 24 h, and the degree of inflammation of lung tissues was assessed with H&E staining (n = 3). **(B)** Lung W/D ratio (n = 3). **(C)** Infiltration of neutrophils into the lung tissues was measured by MPO activity (n = 3). **(D)** The levels of cytokines IL-1β, IL-6, and TNF-α was detected by ELISA (n = 3). **(E)** The miR-106a expression was detected in the lung tissues of LPS treated mice by qPCR (n = 6). U6 snRNA was used as an endogenous control. Data are expressed as mean ± SEM of three independent experiments. *p < 0.05; **p < 0.01 (Student's t-test).

We induced ALI in mice with LPS and excised the lung tissues, then lung injury was assessed by pathological sectioning, the W/D ratio, the MPO assay, and ELISA results. As shown in **Figures 1A**, there were no histopathological changes in the control group. In contrast, obvious lung injury such as infiltration of inflammatory cells, interstitial edema, and alveolar hemorrhage were observed in the LPS group. Then, the W/D ratio, the MPO activity and the levels of IL-1β, IL-6, TNF-α were observably increased in LPS group compared to control group (**Figures 1B-D**). It has been suggested that some miRNAs were induced by LPS, which subsequently regulate LPS-stimulated inflammatory response 31. In order to further investigate whether miR-106a was involved in regulation of inflammation response triggered by LPS, the expression of miR-106a in the lung tissues of ALI mice was measured. qPCR assay showed that miR-106a expression in mice with ALI was suppressed when compared with that of the control group (**Figure 1E**). Therefore, miR‑106a may be a key factor for ALI.

### miR-106a is down-regulated in LPS-stimulated RAW264.7 cells

**Figure 2:**
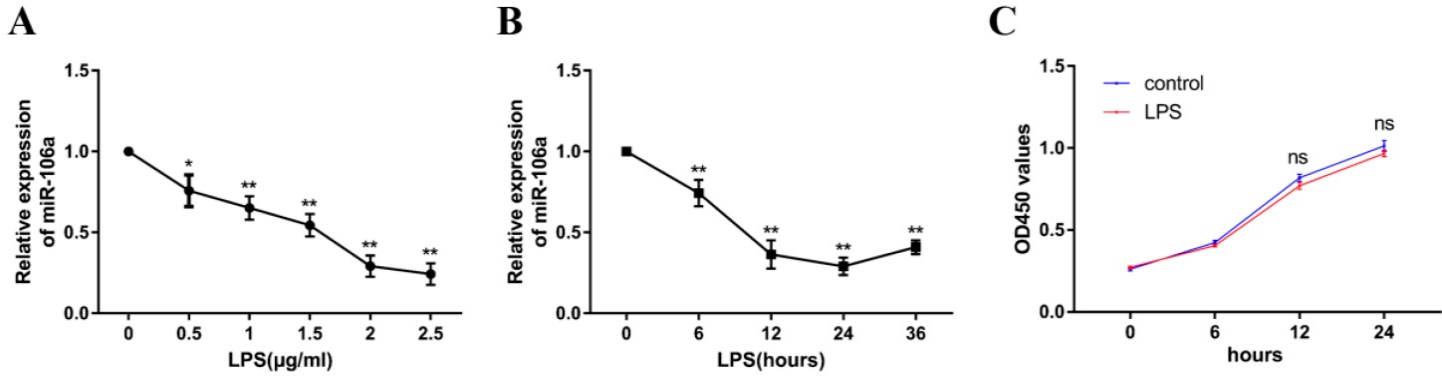
** miR-106a is down-regulated in LPS-stimulated RAW264.7 macrophages. (A)** Macrophages were stimulated with different concentrations of LPS for 24 h. **(B)** Macrophages were stimulated with 2 µg/mL LPS at different times as indicated. Cells were harvested, and miR-106a expression was measured by qPCR. The relative expression of miR-106a was normalized to U6 snRNA. **(C)** The viability of macrophages after treatment with LPS (2 µg/mL) was assessed using a CCK-8 assay kit. Data are expressed as mean ± SEM of three independent experiments. *p < 0.05; **p < 0.01 (Student's t-test).

To explore the mechanism of miR-106a in LPS-induced inflammation in ALI, the level of miR-106a in LPS-induced RAW264.7 cells was also detected. The expression level of miR-106a was dramatically decreased upon LPS treatment, and the down-regulation of miR-106a expression showed a dose-dependent manner (**Figure 2A**). Moreover, we also measured miR-106a expression at different time points of LPS stimulation. The down-regulation of miR-106a levels upon LPS stimulation showed a time-dependent manner and reached a nadir at 24 h (**Figure 2B**). Furthermore, the potential cytotoxicity of LPS at the concentration (2μg/mL) in RAW264.7 cells were evaluated using the CCK-8 assay. The results demonstrated that cell viabilities were not affected by LPS at the dose (2μg/mL) used (**Figure 2C**).These results further verify that miR-106a is involved in LPS-mediated immune responses.

### miR-106a decreases LPS-induced production of inflammatory cytokines

**Figure 3:**
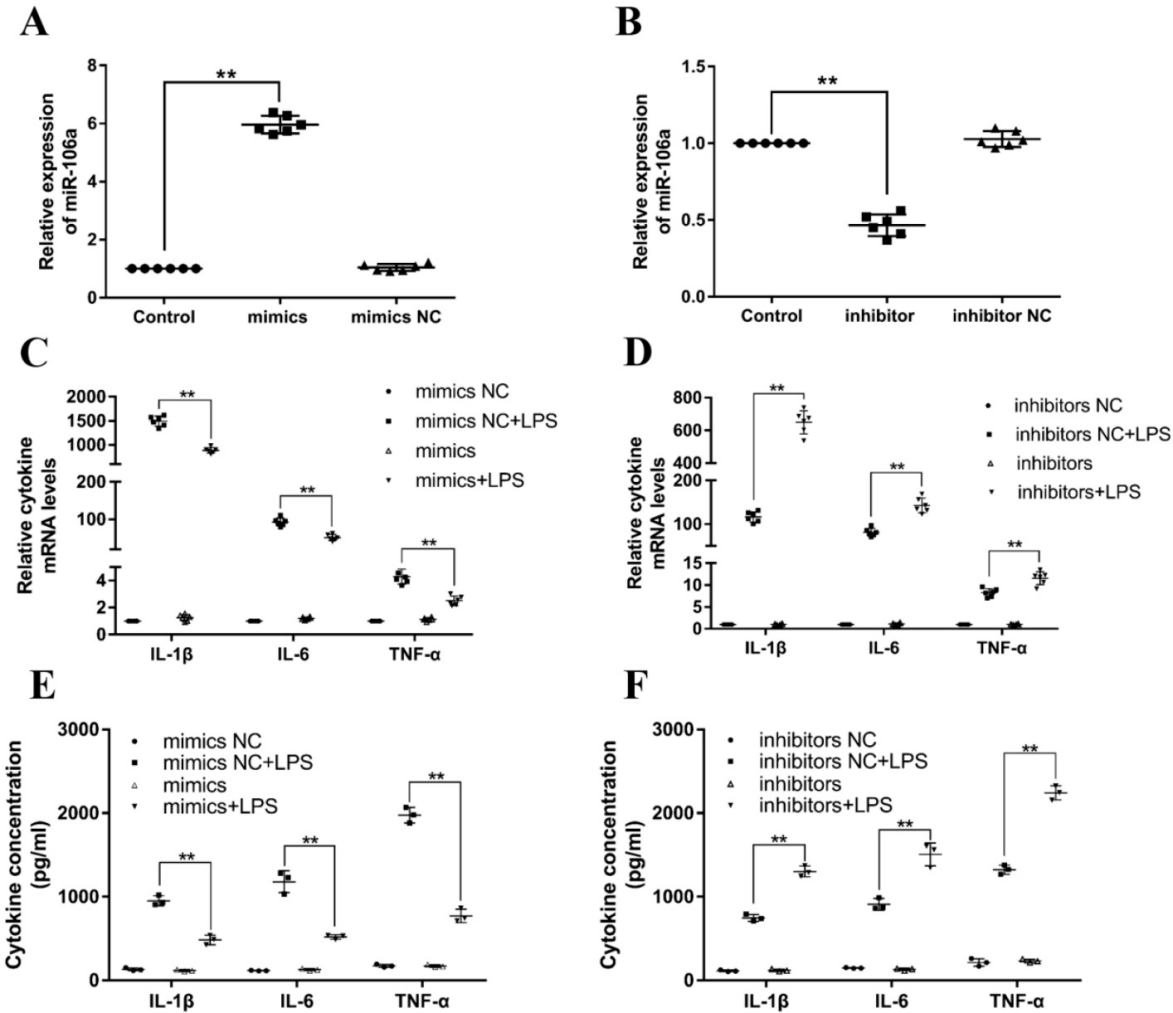
** miR-106a decreases the LPS-induced production of pro-inflammatory cytokines. (A, B)** Macrophages were transfected with miR-106a mimics or inhibitors. At 24 h post-transfection, miR-106a levels were measured by qPCR (n=6). The relative expression of miR-106a was normalized to U6 snRNA. **(C, D)** Cells were transfected with 50 nM miR-106a mimics or 100 nM miR-106a inhibitors for 24 h, and then stimulated with 2 µg/mL LPS for 24 h. The expression of cytokines IL-1β, IL-6, and TNF-α was determined by qPCR (n=6). GAPDH was used as an endogenous control. **(E, F)** The levels of cytokines IL-1β, IL-6, and TNF-α was detected by ELISA (n=3). Data are expressed as mean ± SEM of three independent experiments. *p < 0.05; **p < 0.01 (Student's t-test).

Previous studies have reported that LPS could activate TLR4/NF-κB signaling pathway and subsequently result in the production of inflammatory cytokines, for instance, IL-1β, IL-6, and TNF-α, which all promote the development of ALI 32. In order to investigate the particular role of miR-106a in inflammatory responses, miR-106a mimics or inhibitors were transfected into RAW264.7 macrophages. 24 h after post-transfection, the transfection efficacy was measured by quantitative RT-PCR, and the results verified that transfection with miR-106a mimics or inhibitors led to a conspicuous increase or decrease in miR-106a expression (**Figures 3A, B**). Then, RAW264.7 cells were stimulated with 2 μg/mL LPS for another 24 hours. The mRNA levels and concentrations of cytokines were assessed by qPCR and ELISA. As shown in **Figures 3C-F**, the LPS-induced secretion of pro-inflammatory cytokines were dramatically decreased or increased by over-expression or suppression of miR-106a. It is indicated that miR-106a plays an anti-inflammatory role in the LPS-stimulated inflammatory response.

### miR-106a inhibited LPS-induced activation of NF-κB signaling pathway

**Figure 4:**
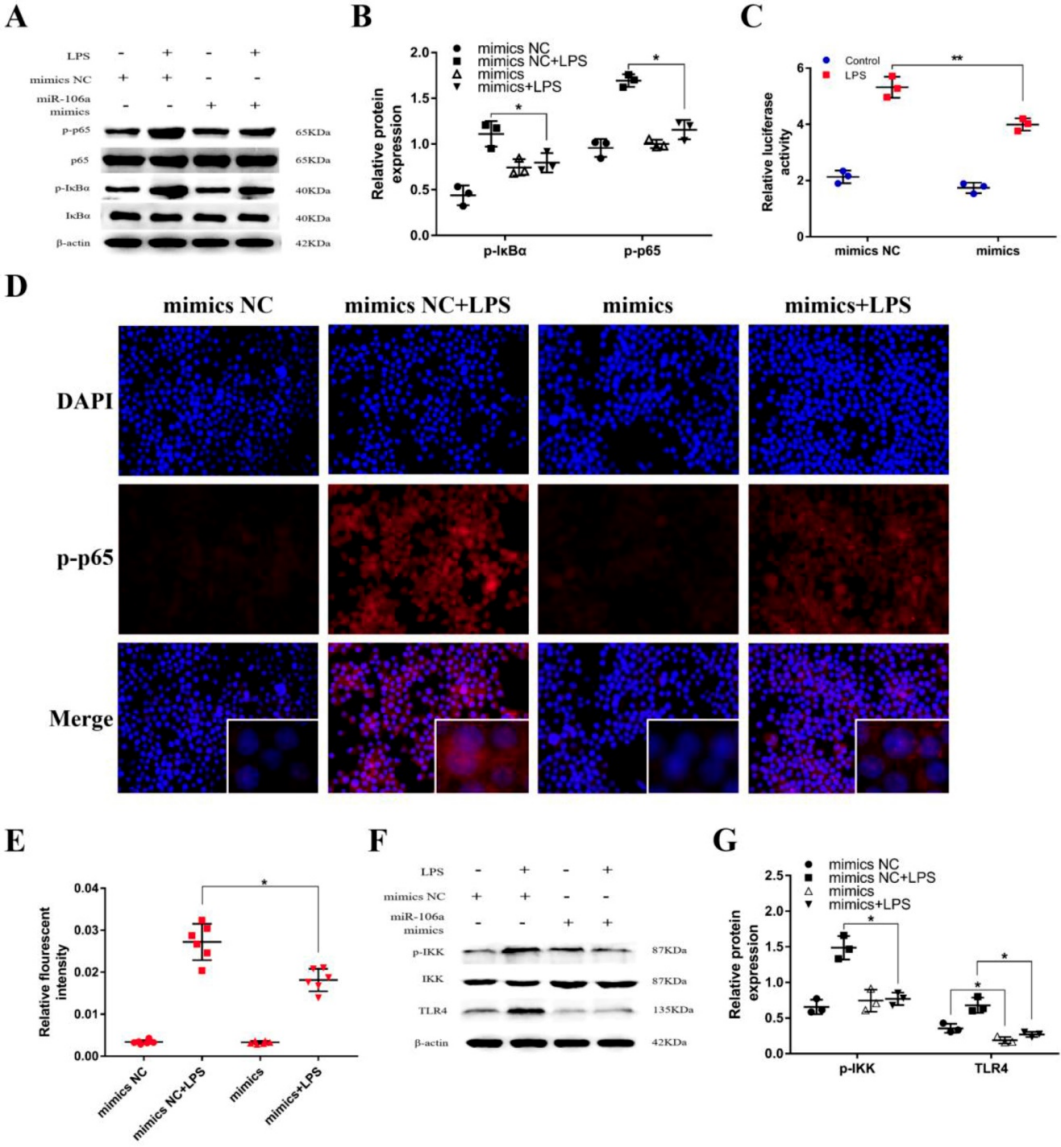
** miR-106a suppressed LPS-induced activation of NF-κB pathway. (A)** Macrophages were transfected with miR-106a mimics or mimics NC for 24 h, then stimulated with 2 µg/mL LPS for 24 h. The protein levels of NF-κB p65 and IκBα were measured by western blotting. β-actin was used as an internal control. **(C)** The NF-κB luciferase activity was measured by dual-luciferase assay (n=3). **(D)** Translocation of the p65 subunit from the cytoplasm into the nucleus was assessed by immunofluorescence staining, scale bar = 50 µm. Blue spots represent cell nuclei, and red spots indicate p-p65 staining. **(E)** The IOD and area of cells were measured by IPP 6.0 software (n=6), and the fluorescence intensity of p-p65 was expressed as IOD/area. **(F)** Cells were treated as **(A)**, and the protein levels of upstream molecules of NF-κB pathway were measured by western blotting. **(B, G)** Gray values of the indicated proteins were measured by Image-Pro Plus (IPP) 6.0 software (n=3). Data are expressed as mean ± SEM of three independent experiments. *p < 0.05; **p < 0.01 (Student's t-test).

It has been generally recognized that the secretion of pro-inflammatory cytokines can be regulated by the NF-κB pathway which is one of the vital signaling pathways of inflammatory response 33. To further inquiry the mechanism function of miR-106a in LPS-induced NF-κB pathway, we detected the protein expression of NF-κB p65 and IκBα by western blot. The results revealed that the protein levels of phosphorylated p65 and IκBα were significantly higher in LPS group than that in control group, while the levels of over-expression miR-106a groups were dramatically reduced (**Figures 4A, B**). In addition, we performed the NF-κB activity assay to elucidate the inhibitory effect of miR‑106a in the TLR4/NF-κB pathway. The results showed that miR-106a suppressed the activity of NF-κB (**Figures 4C**). Moreover, immune-fluorescence results determined that miR-106a mimics caused a decrease in the nuclear translocation of NF-κB p65 after 24 hours of LPS stimulation compared with the control groups (**Figures 4D, E**). Furthermore, we explored the levels of upstream molecules of NF-κB pathway to ascertain the possible target of miR-106a. As shown in **Figures 4F, G**, LPS-induced TLR4 and phosphorylated IKKα/β levels were distinctly inhibited. Besides, the expression of TLR4 protein was also decreased by miR-106a mimics without LPS when compared to the control mimics. These results suggest that miR-106a might inhibit LPS-stimulated activation of NF-κB signaling pathway through decreasing TLR4 expression.

### TLR4 is a molecular target of miR-106a

**Figure 5:**
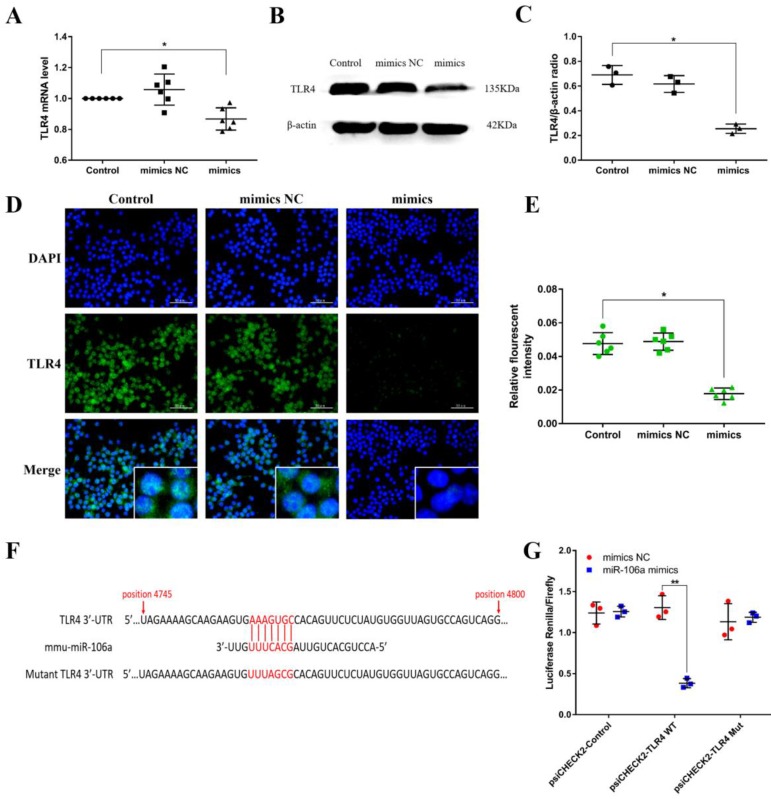
** TLR4 is a molecular target of miR-106a. (A)** Macrophages were transfected with miR-106a mimics or mimics NC for 24 h, the mRNA level of TLR4 was detected by qPCR (n=6). GAPDH was used as an internal control. **(B)** Cells were treated as** (A)**, and the protein level of TLR4 was measured by western blotting. β-actin was used as an internal control. **(C)** Gray values of TLR4 protein were measured by IPP 6.0 software (n=3). **(D)** Immunofluorescence staining was performed to identify the expression of TLR4, scale bar = 50 µm. Blue spots represent cell nuclei, and green spots indicate TLR4 staining. **(E)** The fluorescence intensity of TLR4 (n=6). **(F)** The alignment of miR-106a and TLR4 3'-UTR by computational prediction via the TargetScan and miRanda. **(G)** The dual-luciferase reporter assay was performed in 293T cells (n=3). Cells were co-transfected with the wild- or mutant-type TLR4 3'-UTR luciferase reporter vectors, as well as miR-106a mimics or mimics NC. The ratio of Renilla activity/Firefly activity represents luciferase activity. Data are expressed as mean ± SEM of three independent experiments. *p < 0.05; **p < 0.01 (Student's t-test).

Macrophages were transiently transfected with miR-106a mimics or negative controls, and then we measured the expression of TLR4. According to the results, transfection with miR-106a mimics restrained not only the protein level but also the mRNA level of TLR4, indicating that miR-106a may act on the translational level (**Figures 5A-E**). To identify putative target genes for miR-106a, bioinformatic softwares (TargetScan and miRanda) were performed. As displayed in **Figure 5F**, the analysis has predicted putative binding sites between miR-106a and 3'-UTR of TLR4. In order to further determine whether miR-106a directly binds to TLR4 mRNA, we performed a luciferase reporter assay in HEK293T cells. In brief, 293T cells were transfected with the luciferase-wild type TLR4-3'UTR (WT-3'UTR) or the luciferase-mutant IRAK-3'UTR (Mut-3'UTR) reporter vector, and afterwards treated with miR-106a mimics or control mimics. The results showed that miR-106a dramatically reduced the wild-type-3'UTR but not mutated-3'UTR luciferase levels (**Figure 5G**). These datas indicate that miR-106a suppresses TLR4 expression by directly binding to the 3'-UTR of TLR4 mRNA.

### miR-106a alleviates LPS-induced inflammatory responses through knockdown of TLR4

**Figure 6:**
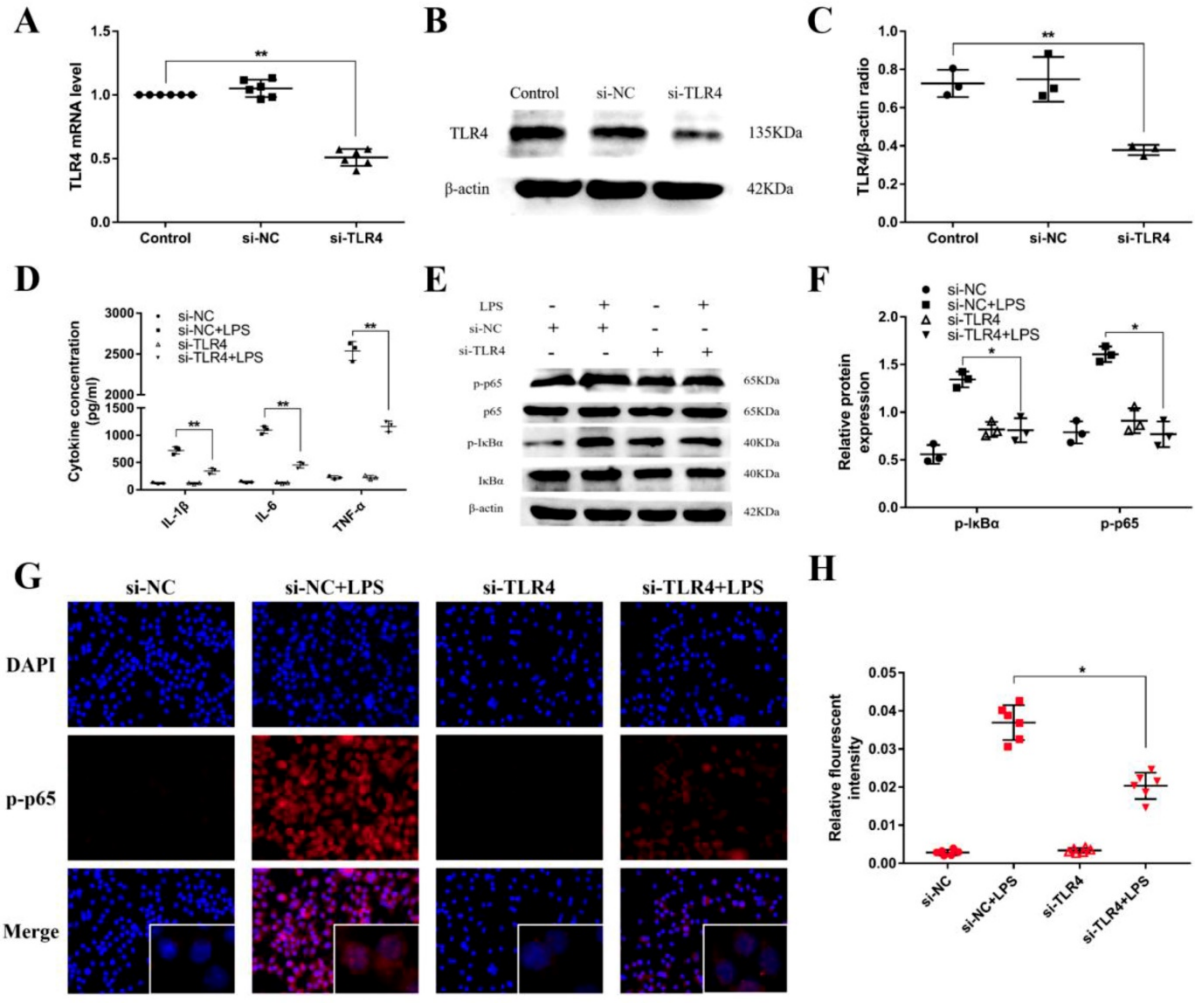
** Knockdown of TLR4 alleviates LPS-induced inflammatory responses. (A)** Macrophages were transfected with the siRNA specific for TLR4 (si-TLR4) or the negative control siRNA (si-NC) at a concentration of 200 nM for 24 h, and the mRNA level of TLR4 was measured by qPCR (n=6). GAPDH was used as an internal control. **(B)** The protein level of TLR4 was determined by western blotting. β-actin was used as an internal control. **(D)** Cells were transfected with 200 nM si-TLR4 or si-NC for 24 h, and then stimulated with 2 µg/mL LPS for 24 h. The levels of cytokines IL-1β, IL-6, and TNF-α were detected by ELISA (n=3). **(E)** The protein levels of NF-κB p65 and IκBα were measured by western blotting. β-actin was used as an internal control. **(G)** Translocation of the p65 subunit from the cytoplasm into the nucleus was assessed by immunofluorescence staining, scale bar = 50 µm. Blue spots represent cell nuclei, and red spots indicate p-p65 staining. **(H)** The fluorescence intensity of p-p65 (n=6). **(C, F)** Gray values of the indicated proteins were measured by IPP 6.0 software (n=3). Data are expressed as mean ± SEM of three independent experiments. *p < 0.05; **p < 0.01 (Student's t-test).

TLR4 has been revealed to play an crucial role in NF-κB activation mediated by LPS 34. For further illuminating the mechanism function of the regulation of miR-106a in LPS-induced inflammatory responses, we used a siRNA for TLR4 (si-TLR4) to knock down the expression of TLR4 in RAW264.7 cells. Subsequently, qPCR and western blot were used to examine the mRNA and protein expression of TLR4. As displayed in **Figures 6A**-**C**, the levels of TLR4 mRNA and protein were obviously reduced after transfection. Besides, the production of the pro-inflammatory cytokines IL-1β, IL-6, and TNF-α were also inhibited (**Figure 6D**). Moreover, knockdown of TLR4 using siRNA vividly inhibited the phosphorylation of IκBα and NF-κB p65, and the above results were consistent (**Figures 6E**-**H**). Overall, these emerging results directly indicate that miR-106a plays an important role in the negative regulation of LPS-stimulated NF-κB activation and pro-inflammatory cytokines production by directly targeting TLR4.

## Discussion

Inflammation is a defense response of the organism against various irritants and pathogens, and also an extremely complex and highly coordinated biological process that restores tissue integrity 35. Under normal circumstances, it is beneficial and provides methods for the organism to deal with various injuries. However, poorly regulated and excessive inflammatory response may become harmful and cause extensive tissue damage, and even systemic dysfunction 36, 37. Acute lung injury (ALI), one of various severe inflammatory diseases, is difficult to treat and has a poor prognosis 38. Although multiple novel pharmacological interventions and treatments have been reported, the mortality rate of ALI is still as high as 50-70% 39. In consequence, it is quite necessary to elucidate the mechanisms in the development of ALI and exploit new strategies for the treatment of ALI. miRNAs are an important class of evolutionarily conserved short (~ 22 nt) endogenous non-coding RNAs, which play a regulatory role in biological processes including proliferation, differentiation, apoptosis, and inflammation 40. A series of previous studies have characterized miRNAs as negative feedback regulator of inflammatory responses, that make sophisticated adjustments to signaling proteins 40, 41.

miR-106a belongs to miR-106 family whose nucleotide sequences are highly conserved in mammals 42. Over the past few decades, miR-106a has been reported to be involved in promoting the proliferation of endometrial cancer RL95-2 cells and inhibiting cell apoptosis 43, in attenuating hyperglycemia induced vascular endothelial cell dysfunction through targeting HMGB1 44, in modulation of tumor cell growth 45. Although some molecular targets are still unknown, it is undeniable that miR-106a is versatile and may have different targets in different circumstances. Even in the same situation, it could own multiple targets as well. To the best of our knowledge, whether miR-106a plays an anti-inflammatory role in the inflammation induced by LPS in ALI remains unclear. Therefore, we elucidated the possible role of miR-106a in LPS-induced inflammatory responses through an *in vivo* ALI model and an *in vitro* inflammatory model using the murine macrophage cell line. In the present study, we showed that miR-106a is significantly down-regulated in the lung tissues of mice and in macrophages in a dose- and time-dependent manner stimulated with LPS. These results indicated that miR-106a may have vital biological functions in LPS-induced inflammation.

Evolutionarily, the innate immune system is the first line of organism defense against pathogen invasion which can identify pathogen associated molecular patterns through pattern recognition receptors, and is the major precipitating factor of inflammatory reactions caused by infection with pathogenic microorganisms 46, 47. TLR4, one of the pattern recognition receptors, is mainly distributed on the surface of multiple immune cells especially mononuclear macrophage, and serves both as signal molecules and receptors of LPS 48, 49. LPS, the main pathogen-associated molecular pattern inducing ALI, interacts with TLR4 receptor on the effector cytomembrane to trigger an intracellular signal transduction system, which can activate the TLR4/NF-κB pathway to initiate gene transcription and produce various pro-inflammatory cytokines, such as IL-1β, IL-6, and TNF-α. In this study, we demonstrated that over-expression or inhibition of miR-106a dramatically decreased or increased the inflammatory cytokines, including IL-1β, IL-6, and TNF-α in RAW264.7 macrophages. NF-κB, an important nuclear transcription factor, plays a key role in a series of inflammatory responses and associated lung damage as well as induction of pro-inflammatory cytokines 50, 51. As expected, the activity of NF-κB signaling pathway followed by LPS stimulation was depressed in macrophages co-transfected with miR-106a mimics. Notably, these datas suggested that miR-106a provides negative feedback to LPS-stimulated inflammation.

In recent years, the function of miRNAs in various diseases has been found to affect TLR4 activation 52, 53. Some studies have indicated that in macrophages, TLR4 plays an important role in triggering NF-κB activation by LPS 34, 54. Much effort has focused on finding TLR4 inhibitors in hopes of developing better anti-inflammatory therapies. Li *et al* showed that inhibition of TLR4 expression in cartilage cut down the seriousness of OA in the rat model 55. More significantly, TLR4 plays a vital part in initiating changes of miRNAs expression in response to invading pathogens 56. Therefore, it is necessary to research the regulatory effects of miRNAs in TLR4 gene expression to further explore therapeutic agents regulating inflammatory diseases. In our study, the analysis indicated that the TLR4 protein level was conspicuously decreased by miR-106a mimics. According to bioinformatics predictions with miRanda and TargetScan, TLR4 is a supposed target of miR-106a. Moreover, the interaction between TLR4 and miR-106a was analyzed by the luciferase reporter assay through the vector containing wild-type TLR4 3'-UTR or mutant-type TLR4 3'-UTR cloned downstream of a fire-fly luciferase reporter. Luciferase expression was obviously decreased when co-transfected miR-106a mimics with WT-3'UTR vector, whereas no significant variation was observed with Mut-3'UTR vector. These data suggest that miR-106a is likely to bind TLR4 mRNA immediately to inhibit its translation. In order to further confirm whether TLR4 acts in the anti-inflammatory effect of miR-106a, si-TLR4 was used to silence TLR4 expression. The results revealed that knockdown of TLR4 improved inflammatory response and LPS-induced NF-κB p65 phosphorylation. Furthermore, we demonstrated that over-expression of TLR4 along with miR-106a followed by LPS stimulation reversed the inhibitory effect of NF-κB signaling mediated by miR-106a. Taken together, regulation of TLR4 is the main mechanism for miR-106a in LPS-stimulated inflammation.

**Figure 7:**
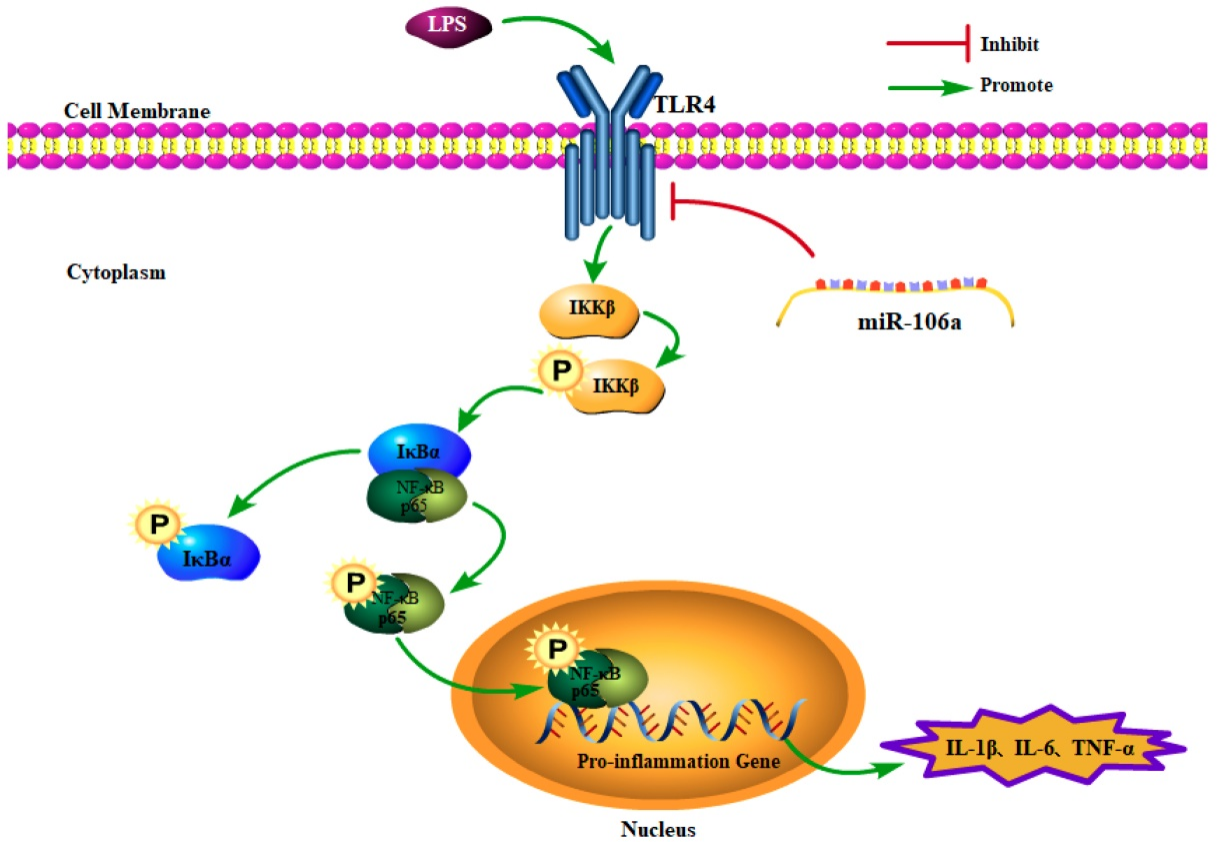
Schematic diagram of signaling pathways related to anti-inflammatory effects of miR-106a on LPS-induced inflammation.

In conclusion, the present study demonstrated that miR-106a was down-regulated in the lung tissues of LPS-induced ALI mice and macrophages. miR-106a inhibited NF-κB signaling pathway and the production of pro-inflammatory cytokines including IL-1β, IL-6, and TNF-α (**Figure 7**). Further study indicated that miR-106a acted by targeting TLR4 expression directly. All these results illustrate that miR-106a may be a negative feedback regulator of LPS-stimulated immune response by suppressing TLR4 expression.

## Figures and Tables

**Table 1:** Sequences for miR-106a and TLR4 siRNA.

miR-106a mimics	Sense	5'-CAAAGUGCUAACAGUGCAGGUAG-3'
	Antisense	5'-ACCUGCACUGUUAGCACUUUGUU-3'
mimics NC	Sense	5'-UUCUCCGAACGUGUCACGUTT-3'
	Antisense	5'-ACGUGACACGUUCGGAGAATT-3'
miR-106a inhibitors	Sense	5'-CUACCUGCACUGUUAGCACUUUG-3'
inhibitors NC	Sense	5'-CAGUACUUUUGUGUAGUACAA-3'
TLR4 siRNA	Sense	5'-CCUCCAUAGACUUCAAUUATT-3'
	Antisense	5'-UAAUUGAAGUCUAUGGAGGTT-3'
siRNA NC	Sense	5'-UUCUCCGAACGUGUCACGUTT-3'
	Antisense	5'-ACGUGACACGUUCGGAGAATT-3'

**Table 2:** Primers Used for qPCR

Name	Primer sequence (5'-3')	GenBank accession number	Product size (bp)
TLR4	TTCAGAGCCGTTGGTGTATCCTCCCATTCCAG GTAGGTGT	NM_021297.2	170
IL-1β	CCTGGGCTGTCCTGATGAGAGTCCACGGGAAAGACACAGGTA	NM_008361.4	131
IL-6	GGCGGATCGGATGTTGTGATGGACCCCAGACAATCGGTTG	NM_031168.1	199
TNF-α	CTTCTCATTCCTGCTTGTGACTTGGTGGTTTGCTACG	NM_013693.3	198
GAPDH	CAATGTGTCCGTCGTGGATCTGTCCTCAGTGTAGCCCAAGATG	NM_001289726.1	124

## Abbreviations

ALI: acute lung injury
LPS: lipopolysaccharide
TLR4: toll-like receptor 4
NF-κB: nuclear factor kappa-B
miR: micro RNA
UTR: untranslated coding region
